# Urban Ecology of Arboviral Mosquito Vectors Along the Kenyan Coast

**DOI:** 10.1093/jme/tjaa136

**Published:** 2021-01-12

**Authors:** Jonathan Karisa, Simon Muriu, Donwilliams Omuoyo, Boniface Karia, Moses Ngari, Doris Nyamwaya, Martin Rono, George Warimwe, Joseph Mwangangi, Charles M. Mbogo

**Affiliations:** 1 Department of Biological Sciences, Pwani University, Kilifi, Kenya; 2 Bioscience Research Centre (PUBReC), Pwani University, Kilifi, Kenya; 3 Center for Geographic Medicine Research Coast, Kenya Medical Research Institute, Kilifi, Kenya; 4 Public Health Department, KEMRI-Wellcome Trust Research Program, Kilifi, Kenya

**Keywords:** Aedes, Culex, arbovirus, ecology

## Abstract

The purpose of this study was to determine the ecology of the common arboviral mosquito vectors in Mombasa, Kilifi and Malindi urban areas of coastal Kenya. Mosquito larvae were collected using standard dippers and pipettes. Egg survivorship in dry soil was evaluated by collecting soil samples from dry potential larval developmental sites, re-hydrating them for hatching and rearing of the eventual larvae to adults. Adult mosquitoes were collected with CDC light traps and BG-Sentinel traps. All blood-fed females were tested for bloodmeal origin. Mosquitoes were screened for arboviruses using RT-qPCR. Overall, the predominant species were *Culex quinquefasciatus* (Say) 72.4% (*n* = 2,364) and *Aedes aegypti* (L.), 25.7%, (*n* = 838). A total of 415 larval developmental sites were identified indoors (*n* = 317) and outdoors (*n* = 98). The most productive larval developmental sites, both indoors and outdoors, were assorted small containers, water tanks, drainages, drums, and jerricans. Overall, 62% (*n* = 18) of the soil samples collected were positive for larvae which were used as a proxy to measure the presence of eggs. The mosquitoes fed on humans (29.8%) and chickens (3.7%). Of 259 mosquitoes tested for viral infection, 11.6% were positive for *Flavivirus* only. The most productive larval developmental sites for arboviral vectors indoors were small containers, water tanks, jerricans, and drums whereas small containers, water tanks, drainage channels, buckets, tires, and water troughs were the productive larval developmental sites outdoors.

Arboviruses are viruses transmitted by a great variety of hematophagous arthropod species, including but not limited to ticks, sand-flies and mosquitoes (Karabatsos 1978). Mosquito-borne viruses are responsible for serious viral disease outbreaks threatening human health and livelihoods especially dengue fever (Ellis et al. 2015, Lutomiah et al. 2016), yellow fever (Ellis et al. 2012), West Nile fever (Kulasekera et al. 2001), and chikungunya fever (Chretien et al.2007, Sang et al. 2008). The emergence and reemergence of arboviral diseases have significantly and negatively impacted on human and animal health (Gubler 2002). An estimated 831 million people are living in mosquito-prone areas and are at risk acquiring arboviral infections, especially so in the tropical and subtropical regions of the world (Woods et al. 2002, Weetman et al. 2018), with a huge burden reported in the sub-Saharan Africa (SSA) (WHO 2019). There exists a gap in knowledge and a need to increase the information and our understanding of the emergence and reemergence of mosquito-borne arboviral diseases and impact on the national public health system.

Key mosquito species in arboviral transmissions are *Aedes aegypti* (Lutomiah et al. 2016) and *Culex quinquefasciatus* (LaBeaud et al. 2011, 2015). *Aedes aegypti* has been incriminated in the transmission of several viruses of public health importance such as Zika virus (ZIKV), dengue fever virus (DENV), chikungunya fever virus (CHIKV), and yellow fever virus (YFV) (Marchette et al. 1969, Diallo et al. 1999, Sang et al. 2008, Amarasinghe et al.2011, Ellis et al. 2012, LaBeaud et al. 2015, Lutomiah et al. 2016, Thangamani et al. 2016, Agha et al. 2017, Konongoi et al. 2018). *Culex quinquefasciatus* is the most dominant and widespread species that have also been incriminated in the transmission of several pathogens. The species is involved in the transmission of West Nile virus (WNV), CHIKV, and Rift Valley fever virus (RVFV) (Goddard et al. 2003, Lutomiah et al. 2011, LaBeaud et al. 2015). Previous studies in Kenya have shown a high abundance of these arboviral vectors and a wide distribution along the Kenyan Coast (Lutomiah et al. 2013, 2016; Ochieng et al. 2013). Despite the envisages high abundance, there is limited routine entomological surveillance and current understanding of the ecology of the arboviral mosquito vectors in the urban coastal landscape.

Several outbreaks of arboviral infections have been reported along the coastal region of Kenya. DENV outbreaks have been reported multiple times in the Kenyan coastal towns of Malindi, Kilifi (Johnson et al. 1982) and most recently in Mombasa (Ellis et al. 2015). Although sporadic, DENV has established its roots in the coastal region of Kenya (Ellis et al. 2015, Konongoi et al. 2016, Karungu et al. 2019). A large outbreak of dengue was in reported in Mombasa in 2013 (Ellis et al. 2015). It led to high morbidity with few fatalities. During entomological surveillance conducted in this period, DENV was isolated from the primary vector, *Ae. aegypti*. Interestingly, the virus was isolated from a pool of male mosquitoes, suggesting transovarial transmission of the virus (Lutomiah et al. 2016). CHIKV fever has been reported in the coastal region of Kenya. In 2003, this virus was reported on the island of Lamu, which led to high morbidity and mortality in that region (Sergon et al. 2008). CHIKV infections have disproportionately affected the people of coastal Kenya. In 2017 through to 2018, over 400 cases were reported, 32 cases were confirmed in the laboratory, whereas the rest were suspected based on the symptoms presented (WHO 2018) The extend of this outbreak has likely been underestimated given the under-reporting of cases and misdiagnosis resulting from insufficient laboratory infrastructure (WHO 2018). Given that there is no treatment and a limited number of available vaccines for most of the arboviral infections, vector control remains the only cost-effective way of preventing arboviral infections. Therefore, understanding the ecology and biology of the common arboviral mosquito vectors in urban coastal Kenya areas through active surveillance would constitute crucial components for effective control of unforeseen outbreaks.

The abundance of mosquito larval developmental sites in the region and inadequate vector control represent major propagating factors for arboviral transmission (Lutomiah et al. 2016). There exist a high diversity and widespread distribution of arboviral mosquito vectors due to the abundance of ideal larval developmental sites (Lutomiah et al. 2016). *Aedes aegypti* breeds in stagnant water, especially in peri-domestic containers such as discarded plastic containers, bottles, coconut husks, old tires, drums, barrels, water storage tanks, obstructed roof gutters, and broken bottles fixed on walls in and around human settlements (Focks and Chadee 1996, Getachew et al. 2015, Lutomiah et al. 2016, Ngugi et al. 2017). *Aedes* mosquito lay eggs during the day in water containing organic material such as decaying leaves of which many eggs adhere to the sidewalls of the containers holding the water. When the water dries up, *Aedes* eggs have the ability to estivate through dry periods in moist/dry soils for several years (Addison et al. 1992; Sota and Mogi 1992a,1992b). When rehy-drated, the eggs hatch into larvae and can be used as a proxy for egg detection and determining the abundance of eggs (Bradley and Travis 1942, Bidlingmayer and Schoof 1956, Silver 2008). The resulting larvae feed upon small aquatic organisms, algae and particles of plant and animal material in water-filled containers. Detection *Aedes* eggs in dry soils from larval developmental sites such as tires, water tanks, tree holes, assorted small containers, further provides information on the preferred oviposition sites. *Culex* is the most common genus with diverse breeding habitats. *Culex quinquefasciatus* have been shown to oviposit in water with high organic content mostly in rice paddies, canals, neglected swimming pools, chambers, drainage, rain pools, ditches, rock pools, septic tanks, tree holes and runoff from agricultural treatment plants (Subra and Mouchet 1984, Irving-Bell et al.1987, Aigbodion et al. 2011). Due to human activities such as water storage, poor disposal of water-holding containers and rapid growth and expansion of urban areas in the tropics greatly increases larval developmental sites for the key arboviral vectors (Aigbodion et al. 2011, Ngugi et al. 2017). There is a need for the development of an effective and sustainable vector control program or strategy against the key arboviral mosquitoes. This ultimately requires knowledge of some aspects of its ecology. The current study was undertaken with over-arching aim of elucidating information on the ecological parameters of arboviral vectors in urban tropical coastal settings of Kenya. The goal was to generate information on essential arboviral transmission risks which would lead to the development of intervention strategies against their mosquito vector populations. Therefore, determining the ecology of key arboviral mosquitoes would provide a way forward in terms of the control of arboviral infections.

## Materials and Methods

### Study Area and Habitat Characterization

**Fig 1:**
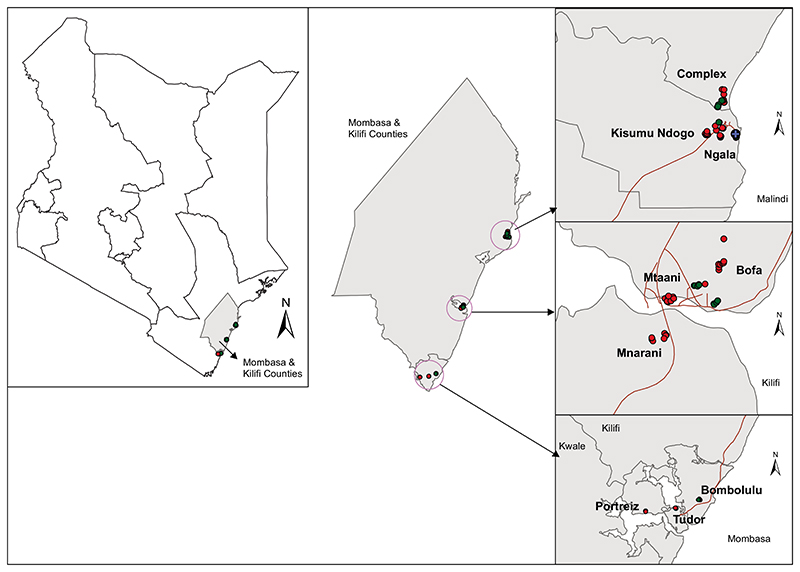
Map showing Mombasa and Kilifi counties where entomological sampling was conducted, i.e., Mombasa (Portreiz, Tudor, and Bombolulu), Kilifi (Bofa, Mtaani, and Mnarani) and Malindi (Ngala, Complex, and Kisumu ndogo) urban sites in coastal Kenya from November 2016 to April 2017.

The study was conducted in three urban coastal areas of Mombasa (4.0435°S; 39.6682°E), Kilifi (3.5107°S; 39.9093°E), and Malindi (3.2192°S; 40.1169°E) (Fig. 1) in Kenya from November 2016 to April 2017, coinciding with the dry season in coastal Kenya. Mombasa urban center (city) has an area of 219.9 km^2^ with a population density of 5,495 persons per square kilometer, whereas Malindi and Kilifi urban centers have an estimated area of about 20 and 8 km^2^, respectively with a population density of approximately 3,500 persons per square kilometer (Kenya National Bureau of Statistics 2019). The Kenyan Coastal region is characterized by dense forests, savanna vegetation, seasonal swamps, dry thorn bushes and diverse plantations interspersed with furrow land. Sisal, coconut, and cashew nut plantations are prominent along the coast although subsistence farming is common in inland areas. The region experiences bimodal rainfall with the long monsoon rains occurring in April to July and the short rains between October and December. The relative humidity ranges from 55 to 65% and the mean annual temperature between 20 to 35°C with an annual rainfall of 750 to 1,200 mm. The counties of Mombasa and Kilifi are characterized by a flat topography. Altitudes range from 0 to 400 m above sea level. The rural areas are mainly inhabited by the Mijikenda and Swahili communities, while urban areas have a mixed population of different Kenyan communities and tourists from around the globe. The major economic activities are tourism, fishing, commercial trade and retail, and service professions, whereas the informal economic sector is comprised of street vendors, sex workers, and tour guide services. These sites are interspersed with commercial, undeveloped, farmed, and residential areas. Houses consist of concrete or mud walls with iron sheets or palm leaf (Makuti) roofing. Most households in the rural areas keep goats, chickens, cats, ducks, dogs and cattle whereas those in the urban setting, due to lack of space, keep very few or no livestock at all. Animals found in the urban areas include chicken, pets (dogs, cats) and mice. Wild birds, especially ravens (*Corvus corax*), were abundant in all the study sites. In each site, three residential estates were selected for larval and adult mosquito sampling.

## Mosquito Larval Sampling

### Larval Sampling

Mosquito larval sampling was done in three randomly selected residential estates in each of the three urban study sites of Mombasa, Kilifi, and Malindi. Potential *Aedes* and *Culex* mosquito breeding habitats inside and outside houses were identified and sampled for mosquito larvae. The indoor and outdoor water containers were identified and visually checked for mosquito larvae and pupae.

Depending on the habitat size and type, mosquito sampling was done using standard dippers (3*5*0 ml): *5*–20 dippers per container or by pipetting. The mosquito samples from each habitat were placed in individually labeled whirl-packs in a cooler box and transferred to the laboratory for further processing.

### Larval Habitat Classification

Drums were defined as cylindrical containers with a capacity of between 50 and 200 liters while water tanks were defined as any water storage container with 200–1,000 liters of water storage capacity. The assorted/discarded small containers comprised small plastic/metallic containers of less than 10 liters water-holding capacity (Focks 2004, Focks and Alexander 2017).

### Sampling for *Ae. aegypti* Egg Survivorship in the Dry Substrate

Sampling of *Aedes* eggs was conducted in potential *Aedes* breeding habitats identified in the residential estates in Kilifi and Malindi urban study sites. During sampling, the dry soil or substrate from the potential breeding habitats was sampled by scooping a handful (about 50 g) of the soil or substrate with a spatula, placed in whirl-paks and transferred to the laboratory for further processing in the insectary.

### Adult Mosquito Sampling

Adult mosquitoes were sampled using Biogent (BG) Sentinel traps (Biogents AG, Germany) (outdoors) and standard Centre for Disease Control light traps (John W. Hock Company, Gainesville, FL) (indoors). Light trapping was conducted in December and January whereas BG trapping was done in March. The surveys were conducted successively (from one site to the next). Indoor and outdoor sampling was to be done simultaneously, but due to resource logistics, we were limited to successive sampling. BG-Sentinel traps were primarily deployed for surveillance of adult *Ae. aegypti* (Maciel-de-Freitas et al. 2006). Twenty-seven BG-sentinel traps baited with carbon dioxide (about 3 kg dry ice per trap per sampling day) were randomly set outdoors from 0600 to 1700 hours, in each of the three-urban settings. The traps were systematically set at ground level at intervals of 100 m from each other. The BG traps were powered by 12 volts, 12-amp DC battery.

The CDC light traps were set up between 1800 hours and left to run overnight and collected at 0600 hours the following morning. Forty traps were set in selected houses in Mombasa and Malindi while 30 traps were set in Kilifi. The power source for the traps was 6 volts, 12-amp DC battery. The mosquito samples collected were transported to the laboratory in dry form in a cool box for further processing.

## Laboratory Sample (Mosquito) Processing

### Egg Processing

The soil or substrate sample collections were placed in individually labeled basins and one liter of chlorinated tap water added and allowed to settle as previously described (Bradley and Travis 1942, Bidlingmayer and Schoof 1956, Silver 2008). This was performed under insectary conditions with temperature for larvae breeding and rearing maintained at between 32 and 34°C and in the adult breeding room at between 26 and 28°C while relative humidity of 70–80% for both larvae and adults with 12:12 (L:D) h. The basins were monitored daily for eggs to hatch into first instar larvae. Larvae typically fed on algae and other microscopic organisms that are naturally found in water. The resultant larvae were reared to pupae that were enumerated, recorded and transferred to pupal cages for adult emergence. All the soil/substrate samples were monitored for 2 wk and those that did not produce any larvae were regarded as negative samples. The emergent adults were enumerated and morphotyped using identification keys (Edwards 1941).

### Larval Rearing

In the laboratory, the larvae were grouped as early (L1 and L2) or late (L3 and L4) stage and reared in plastic basins using water obtained from the site of larval collection. Larval development was monitored daily and all pupae harvested using a Pasteur pipette, placed in pupal cups in mosquito cages for adult emergence. Larvae fed on algae and other microscopic organisms that are naturally found in water but were in some cases supplemented with Tetramin baby fish (Aquarium shop, Germany). The emerging adult mosquitoes were fed with a 10% glucose solution presented on cotton pads until identification. Temperature for larvae breeding and rearing was maintained at between 32 and 34°C and in the adult breeding room at between 26 and 28°C while relative humidity of 70–80% for both larvae and adults with 12:12 (L:D) h.

### Adult Mosquito Processing and Identification

All adult mosquitoes from the field and insectary were killed by placing them at -20°C for 10 min. The mosquitoes were then sorted from other arthropods and morphologically identified to species level using identification keys as developed by Edwards (1941). All the samples were preserved in 1.5 ml cryogenic vials at -80°C for arbovirus detection.

### Blood Meal Analysis

All blood-fed mosquitoes collected from the field were cut transversely at mid-section to separate the head and thorax from the abdomen. The abdomen was placed in a labeled vial while the rest were preserved at -80°C for arboviral detection. Blood meal analysis was done using an Enzyme-Linked Immunosorbent Assay (ELISA) as described previously (Beier et al. 1988, Mwangangi et al. 2003, Muturi et al. 2008). The positive controls were serum from human, bovine, goat and chicken, whereas PBS was used as negative control. Results were visually evaluated through color change (homogenous greenish-blue color for positive and clear for negative samples).

### RNA Extraction

All adult mosquito samples from larval, habitat substrate or soil, and adult collections were processed on a chill table by pooling them (1 to 25 mosquitoes per pool) by site, method of collection, species, and sex. RNA was extracted from mosquito samples using the Trizol-LS - Chloroform extraction method (Chomczynski and Sacchi 1987, 2006). Briefly, the mosquito samples (pools of intact mosquitoes) were homogenized by crushing using sterile pestles in 1 ml of TRIzol reagent followed by the addition of 0.2 ml chloroform to the homogenate and vortexed for 30 s. The resultant homogenate was incubated for 2–3 min centrifuged at 12,000 rpm for 15 min at 4°C.

The aqueous phase was then transferred to an Eppendorf tube and the RNA precipitated by mixing with 0.5 ml isopropanol followed by incubation at room temperature for 10 min. The mixture was then centrifuged at 12,000 rpm for 10 min at 4°C and the supernatant removed before washing the pellets with 1 ml of 75% ethanol by flicking followed by centrifugation at 7,500 rpm for 10 min at 4°C. The supernatant was removed, and the pellet air-dried. The final RNA pellet was dissolved in 50 μl of nuclease-free water at room temperature and stored on ice or frozen at -80°C for subsequent arbovirus screening.

### Arbovirus Screening

The extracted RNA was tested using primers targeting *Flavivirus*, *Alphavirus*, and *Phlebovirus* arboviral genera. Virus detection and amplification were done using the QuantiFast Multiplex (Qiagen) RT–PCR + R kit in conjunction with primers and probes designed for generic amplification of *Flavivirus* nonstructural *5* genes (NS5), *Alphavirus* nonstructural protein 4 (NSP4) gene and *Phlebovirus* primers targeting the Large (L) and small (S) segments. The protocol for *Flavivirus*, *Alphavirus*, and *Phlebovirus* assay have been described elsewhere (Mwaengo et al. 2012, Patel et al. 2013, Giry et al. 2017). DENV specific assay was performed to all samples that tested positive for *Flavivirus*. The ABI 7500 real-time PCR (Applied Biosystems, United States) was used for amplification.

### Data Management and Analysis

Data collected were entered into Microsoft Excel and analyzed in Stata statistical package (StataCorp 2011. *Stata Statistical Software: Release 11*. College Station, TX: StataCorp LP) (StataCorp 2011). The mean number of immature mosquitoes indoors and outdoors was calculated, and the difference compared within each site using a *t*-test. Chi-square was used to measure the association between site, sex, and species variation regards to flavivirus positivity. Statistical differences between and among groups were deemed significant at *P* < 0.05. The larval mosquito infestation indices were calculated as House Index (HI)—the percentage of houses positive with immature mosquitoes, Container Index (CI)—the percentage of water-holding containers in which mosquito breeding is occurring and Breteau Index (BI)—the number of positive containers per 100 houses as previously described (Sanchez et al. 2006, Lutomiah et al. 2016). Shannon diversity index (*H*) was used to characterize species diversity in the three study sites in urban coastal Kenya as previously described (Muturi et al. 2006). The mean (±SD) number of mosquitoes collected per method of collection in each site was calculated.

## Results

### Larval Habitats Diversity and Productivity

A total of 415 mosquito breeding habitats were identified inside (317) and outside (98) houses. Out of these, 168 (40.*5*%) were found in Kilifi, 114 (27.*5*%) in Malindi and 133 (32.04%) in Mombasa (Table 1). Fourteen different larval developmental sites were identified and sampled in the three study sites. Overall, the most prevalent breeding habitats in the three sites were jerricans (66.9%), followed by water tanks (10.6%), small containers (6.8%), and drainage channels (6.0%). Other larval developmental sites encountered in small numbers were buckets, basins, ditches, water troughs, flower-pots, swimming pools, chambers, and earthen water pots (Table 1).

Overall, the most productive habitats indoors were drums, small containers, jerricans and water tanks whereas for outdoors the most productive containers were drainage channels, small containers, tires, water tanks, jerricans, and water troughs (Table 1). There was a significant association between habitat type and immature productivity (*P* < 0.001). Productivity, in this case, can be defined as the efficiency of larval developmental sites to produce larvae. A total of 18 larval developmental sites in Kilifi (6% indoors, 31% outdoors) were positive for mosquito immature stages and significant difference in the density of immatures between indoor and outdoor (*P* < 0.05) existed. The most productive indoor larval developmental sites in Kilifi were small containers, water tanks, and jerricans whereas outdoors were drainage channels, small containers and water tanks (Table 1). In Malindi, 12 habitats (2% indoors, 22% outdoors) were found to be positive for mosquito immatures and there was no significant difference between indoor and outdoor positive habitats (*P* > 0.05). In Malindi, larval indoor developmental was restricted to jerricans. The most productive larval developmental sites outdoors were water tanks, jerricans, small containers and the least were tires (Table 1). In Mombasa, 18 habitats (13% indoors, 15% outdoors) were found to be positive for mosquito immatures (Table 1). There was no significant difference in the density of immatures between indoor and outdoor (*P* > 0.05). In Mombasa, the most productive indoor larval developmental sites were water tanks, drums and jerricans whereas for outdoor, water tank was the most productive followed by tires and the least were drainage channels (Table 1).

### Species Composition in Larval Habitat Collection

Overall, 889 adult mosquitoes belonging to two genera (*Aedes* and *Culex*) emerged from the larval population collected. The majority were *Ae. aegypti* (85.3%) and the rest being *Cx. quinquefasciatus* (12.6%), *Aedes vittatus* (Bigot, 1861) (1.1%), and *Culex zombaensis* (Theobald, 1901) (1.0%). Indoor immatures resulted in purely and exclusively *Ae. aegypti* whereas outdoor had both *Ae. aegypti* and *Cx. quinquefasciatus*.

### Larval Infestation Indices

Overall, 55 houses (30 in Kilifi, 11 in Malindi, and 14 in Mombasa) were sampled from the three sites for mosquito larval developmental sites. Of these houses, 18 had containers that were positive for *Ae. aegypti* immatures, giving an overall House Index (HI) of 32.7%. A total of 317 containers were inspected indoors giving an overall Container Index (CI) of 8% and Breteau Index (BI) of 45.4. Mombasa had the highest indices (HI of 71.4, CI of 13.3 and BI of 107.1) compared to Malindi and Kilifi (Table 2).

### Mosquito Egg Survivorship in Dry Habitats

A total of 29 dry larval development site substrate/dry larval development site soil samples were collected from the water tank (*n* = 2), small container (*n* = 1), tires (*n* = 16) and flowerpots (*n* = 10). Overall, 62% (*n* = 18) of the soil samples collected from the two sites (Kilifi and Malindi) were positive for larvae. Five hundred six adult mosquitoes resulted from the larvae reared from the dry larval development site substrate. Three *Aedes* species, *Ae. aegypti* (98.4%), *Aedes hirsutus* (Theobald, 1901) (1.4%), and *Ae. vittatus* (0.2%), were identified (Table 3).

### Adult Mosquito Distribution and Abundance

The relative abundance of adult mosquitoes collected indoors and outdoors by the Biogents Sentinel (BG) traps and Light traps (LT) is summarized in Table 4. Overall, 3,264 mosquitoes belonging to three genera (*Culex*, *Aedes*, and *Anopheles*) and 10 species were collected. *Culex quinquefasciatus* (2,364) and *Ae. aegypti* (838) were the most common species, and the least were *Aedes mcintoshi* (Huang, 1985), *Aedes pembaensis* (Theobald, 1901), and *Culex annulioris* (Theobald, 1901) (*n* = 1). *Culex quinquefasciatus* were mostly collected indoors (*n* = 2,140) compared to outdoors (*n* = 260) while more *Ae. aegypti* were captured outdoors (*n* = 816) compared to indoors (*n* = 22) (Table 4). Malindi had the highest number of mosquitoes collected by CDC light traps (28.9 ± 29.4) followed by Mombasa (18.4 ± 38.1) and Kilifi (9.2 ± 10.0). Kilifi had the highest number of mosquitoes collected by BG-Sentinel traps (19.6 ± 36.7) followed by Malindi (16.9 ± 19.9) and Mombasa (4.1 ± 5.1) (Table 1). Shannon diversity index (*H*) and evenness (*EH*) of mosquito species indicated a higher species diversity in Kilifi (*H* = 0.840) compared to Malindi (*H* = 0.662) and Mombasa (*H* = 0.385). Mosquitoes were evenly distributed in Kilifi (*EH* = 0.469) compared to Malindi (*EH* = 0.370) and Mombasa (*EH* = 0.215).

### Blood Meal Origins

Out of the 161 blood-fed females tested by ELISA for host bloodmeal origins, 91% (*n* = 146) were from *Culex* and the rest were *Aedes* (9%, *n* = 15) species (Table 5). The samples were tested against four bloodmeal source/antisera namely: bovine, chicken, goat, and human. The majority of the samples could not be identified (66.5%) for bloodmeal sources, of those identified 29.8% had fed on the blood of human origin while 3.7% had consumed chicken blood. None of the mosquitoes had fed on goat or bovine. Given that sampling was restricted in the urban areas where minimal farming is practiced and livestock absent, this was not unexpected. The mosquitoes analyzed comprised of *Cx. quinquefasciatus* (*n* = 143), *Ae. aegypti* (*n* = 15), and *Cx. univittatus* (*n* = 3). The majority (*n* = 140) of the 161 blood feds were collected indoors (Table 5).

### Arboviruses Diversity in Mosquitoes

Virus was detected in 11.6% of the 259 pools screened against the three viral genera. Overall, the pools consisted of 129 *Ae. aegypti* pools and 130 *Cx. quinquefasciatus* pools. The overall positive pools (*n* = 30) were only positive for *Flavivirus* and none for either *Phlebovirus* or *Alphavirus. Ae. aegypti* had a significantly higher (χ^2^ = 18.4398, df =1, *P* = 0.001) proportion of virus-positive pools (87%, *n* = 26) compared to *Cx. quinquefasciatus* (13%, *n* = 4).

*Ae. aegypti* had 129 (60 females and 69 males) pools screened, 20.1% (*n* = 26) of the pools were positive for *Flavivirus*. There was a site to site variation in terms of *Flavivirus* positivity in the mosquito pools from the three sites (χ^2^ = 14.2292, df = 2, *P* = 0.001). In Kilifi, 18 pools (five for females and 13 for males) of *Ae. aegypti* tested positive for *Flavivirus*. In Mombasa, only three pools were positive for *Flavivirus* and comprised of *Ae. aegypti* only (one pool for females and two for males). In Malindi, five female pools of *Ae. aegypti* tested positive for *Flavivirus*. There was no significant difference between male and female positivity (χ^2^ = 0.2697, df = 1, *P* = 0.604) (Table 6). *Culex quinquefasciatus* had only 4 pools which tested positive for *Flavivirus*, 1 pool from Kilifi, 3 from Malindi and none from Mombasa (Table 6). All *Flavivirus* positive samples were negative for DENV.

## Discussion

Diverse larval developmental sites were reported outdoors, though limited in numbers and corroborate with the results of Ngugi et al. (2017) on the distribution of the breeding habitats. Discarded tires, drums, water tanks, buckets, small domestic containers, water troughs, and jerricans have been identified to be the key larval developmental sites of *Ae. aegypti* (Focks and Chadee 1996, Midega et al. 2006, Sanchez et al. 2006, Getachew et al. 2015, Ngugi et al. 2017). Low indoor larval productivity in our study sites can also be attributed to human activities related to the use of domestic water storage devices. The fact that the majority of the indoor containers are in regular use curtailed the life cycles of immature mosquitoes.

Additionally, in most cases, the indoor containers are usually covered because the water is used for cooking and drinking thus limiting the opportunity of gravid mosquitoes to oviposit. They are, therefore, less likely to harbor mosquito immatures (Ngugi et al. 2017). High outdoor larval productivity was reported in all three study sites. This could have been attributed by poor disposal of items which when filled with water become potential larval developmental sites. Local suppliers for tri/bi/motorcycles and other vehicles has led to the poor disposal of unused tires. Water tanks and small containers were mostly found in construction sites and commercial flower gardens as water-holding containers. This study shows that water tanks are suitable breeding habitats for mosquitoes in indoors and outdoors. During the long dry season, drums and water tanks could play a significant role by acting as larval developmental sites of *Ae. aegypti* as they are used to store water and this was consistent with other studies (Ngugi et al. 2017). Tires, considered an important breeding sites for *Ae. aegypti* (Lutomiah et al. 2016) produced only a small proportion of the larvae collected outdoors. This could be attributed to the period of the collection as sampling was done during the dry period. Additionally, soil samples collected in this study were mostly from tires and most of the positive samples were from tires. This further supports the preference of *Aedes* mosquitoes to oviposit in tires (Lutomiah et al. 2016, Ngugi et al. 2017). *Aedes* eggs are usually laid/placed on the walls of the containers which might fall off to the bottom when the water in the container dries out, thus making it possible to collect eggs from the dry soil that was inside the potential LDS. Eggs of some species in the genus *Aedes* can remain dormant and viable in dry soil for a long period, as the study area had not experienced rains for more than 8 mo and corroborates with previous studies (Sota and Mogi 1992a).

The current study further investigated the larval indices which are not only used to determine the preferred larval developmental sites but also the impact of vector control intervention specifically the container-based *Ae. aegypti* control intervention (Focks and Chadee 1996, Focks 2004, Midega et al. 2006, Sanchez et al. 2006, Lutomiah et al. 2016, Focks and Alexander 2017). Poor water storage could have resulted in high larval indices. This study enrolled a few households (*55* house and 317 containers) thus we cannot draw a conclusion and cannot be compared with the WHO indices. This calls for detailed research to be conducted to establish the larval indices during the dry and wet seasons in the coastal region of Kenya.

The present study showed that potential arbovirus mosquito vectors are abundant in the coastal region, although in varying abundances. Variation in arboviral mosquito density and species richness was observed in the three sites. This could be due to the observed differences in the diversity of aquatic habitats among the three sites. Kilifi had more productive habitats compared to Malindi and Mombasa, thereby supporting diverse mosquito species. Previous studies have reported that habitat type diversity is directly proportional to mosquito species richness (Gleiser et al. 2002, Muturi et al. 2006). *Aedes aegypti* and *Cx. quinquefasciatus* were the most abundant mosquito species in urban areas of coastal Kenya could potentially be contributing to the current outbreaks of arboviral infections of public health and veterinary importance in the region. *Flavivirus* was isolated from both *Ae. aegypti* and *Cx. quinquefasciatus* indicates that these mosquitoes are vectors of an array of arboviruses (Crabtree et al. 2009; Kent et al. 2010; LaBeaud et al. 2011; Lutomiah et al. 2011, 2016). Virus isolates from male mosquito pools suggests transovarial transmission (Lutomiah et al. 2016). The risk of transmission DENV, CHIKV, WNV, and YFV will be high in the absence of effective vector control, therefore entomological surveillance should be conducted in this region to determine the significance of these viruses in the local vector populations. *Phleboviruses* and *Alphaviruses* were not detected in the mosquito samples tested in the study samples. Similarly, all *Flavivirus* positive were specifically screened for DENV, and none of the pools turned positive. This does not mean that these viruses are absent in the region, but rather could mean low infection rates or the viruses were not in circulation during the time of sampling. DENV which belongs to the genus *Flavivirus* has established its roots in the coastal Kenya and is reported in almost each other year, therefore it was worthwhile to specifically screen for this virus to deduce if it was in circulation during the sampling period.

Mosquito (vector) blood-feedings provide a crucial component in understanding the transmission dynamics of pathogens including viruses and parasites. A larger portion of the blood meals could not be identified. This could have been as a result of lack of antisera to test for all the hosts that have been reported in the study areas. Other potential hosts present in the areas were dogs, cats, wild birds, and rodents, although logistical and resource limitations restricted ELISA tests against them despite these animals being known to be important bloodmeal sources for mosquitoes (Kamau et al. 2003). It was shown that none of the mosquitoes had fed on goats or bovine although these animals are few or absent in the urban settings. *Culex quinquefasciatus* showed a preference for both humans and chicken whereas *Ae. aegypti* fed on humans though in low numbers (Muturi et al. 2008). Being efficient vectors of various arboviruses, these mosquito species can play a significant role in the transmission of arboviruses in the coastal region of Kenya.

In conclusion, the study highlights the key larval developmental sites, diversity, abundance, distribution, trophic preferences and the infection status of key arboviral vectors in the coastal region of Kenya. This highlights the potential for the emergence of arboviruses in the coastal populations. There is a need to map the countrywide distribution and abundance of culicine mosquitoes. Assessment of the infection status and bloodmeal sources will provide key indicators on the transmission dynamics of arboviruses of public health importance in Kenya.

## Figures and Tables

**Table 1:** Summary of the habitat surveyed and their productivity for indoor and outdoor locations in Kilifi, Malindi and Mombasa urban sites in coastal Kenya from November 2016 to April 2017 (LDS = larval developmental sites)

LDS type	Kilifi						Malindi						Mombasa					
	Indoor			Outdoor			Indoor			Outdoor			Indoor			Outdoor		
	No. of LDS surveyed (positive)	Mean larvae	Mean pupae	No. of LDS surveyed (positive)	Mean larvae	Mean pupae	No. of LDS surveyed (positive)	Mean larvae	Mean pupae	No. of LDS surveyed (positive)	Mean larvae	Mean pupae	No. of LDS surveyed (positive)	Mean larvae	Mean pupae	No. of LDS surveyed (positive)	Mean larvae	Mean pupae
Small containers	9(1)	10	0	10(5)	108	46	0(0)	0	0	8(2)	27	0	0(0)	0	0	1 (0)	0	0
Drums	0(0)	0	0	0(0)	0	0	0(0)	0	0	0(0)	0	0	7(6)	43	3	0(0)	0	0
Water tanks	24(6)	9	1	3(1)	15	0	3(0)	0	0	3(2)	74	70	9(3)	44	5	2(1)	41	0
Jerricans	104 (2)	7	1	0(0)	0	0	62(1)	26	4	19 (1)	50	0	91 (6)	28	1	2(0)	0	0
Buckets	0(0)	0	0	0(0)	0	0	0(0)	0	0	3(2)	58	50	2(0)	0	0	0(0)	0	0
Basins	0(0)	0	0	0(0)	0	0	0(0)	0	0	1 (0)	0	0	3(0)	0	0	0(0)	0	0
Drainage channels	2(0)	0	0	9(3)	87	4	0(0)	0	0	6(0)	0	0	0(0)	0	0	8(1)	17	4
Ditches	0(0)	0	0	1 (0)	0	0	0(0)	0	0	1 (0)	0	0	0(0)	0	0	2(0)	0	0
Tires	0(0)	0	0	3(0)	0	0	0(0)	0	0	1 (1)	20	0	0(0)	0	0	5(1)	30	0
Water troughs	0(0)	0	0	0(0)	0	0	0(0)	0	0	3 (3)	36	0	0(0)	0	0	0(0)	0	0
Flowerpots	0(0)	0	0	0(0)	0	0	0(0)	0	0	3(0)	0	0	0(0)	0	0	0(0)	0	0
Swimming pools	0(0)	0	0	0(0)	0	0	0(0)	0	0	1 (0)	0	0	0(0)	0	0	0(0)	0	0
Chambers	0(0)	0	0	3 (0)	0	0	0(0)	0	0	0(0)	0	0	0(0)	0	0	0(0)	0	0
Water pots	0(0)	0	0	0(0)	0	0	0(0)	0	0	0(0)	0	0	1 (0)	0	0	0(0)	0	0
Total	139 (9)	26	2	29 (9)	210	50	65(1)	26	4	49 (10)	216	120	113 (15)	115	9	20 (3)	88	4

**Table 2:** Indoor site-specific House index (HI), Container index (CI), and Breteau index (BI) in Kilifi, Malindi, and Mombasa urban sites in coastal Kenya from November 2016 to April 2017

Sampled site	No. houses of sampled houses	No. of positive houses	HI	No. of wet habitats	No. of positive habitats	CI	BI
Kilifi	30	7	23.33	139	9	6.47	30.00
Malindi	11	1	9.09	65	1	1.54	9.09
Mombasa	14	10	71.43	113	15	13.27	107.14
Overall	55	18	32.73	317	25	7.89	45.45

**Table 3:** Summary of the soil samples collected from different containers, positive habitats and the mosquito species that emerged from Kilifi and Malindi urban sites in coastal Kenya from November 2016 to April 2017 (% +ve = percentage or proportion of larval developmental sites [LSD] positive)

Site	LDS	No. of LDS (% +ve)	Mosquito species	Total adults emerged
Kilifi	Tires	7 (57)	*Ae. aegypti*	319
			*Ae. hirsutus*	7
			*Ae. vittatus*	1
	Flowerpots	3 (66)	*Ae. aegypti*	2
	Water tanks	2 (0)	-	
Malindi	Small container	1 (100)	*Ae. aegypti*	16
	Tires	9 (77)	*Ae. aegypti*	79
	Flowerpots	7 (71)	*Ae. aegypti*	82

**Table 4:** The relative abundance of mosquito species collected using light trap (indoor) and Biogents (BG) sentinel trap (outdoor) from in Kilifi, Malindi and Mombasa urban sites in coastal Kenya from November 2016 to April 2017

Mosquito species	Light trap (indoor)			BG-sentinel trap (outdoor)			Total
	Kilifi (30 traps)	Malindi (40 traps)	Mombasa (40 traps)	Kilifi (27 traps)	Malindi (27 traps)	Mombasa (27 traps)	
*Ae. aegypti*	3	9	10	306	446	64	838
*Ae. hirsutus*	3	0	0	0	0	0	3
*Ae. mcintoshi*	1	0	0	0	0	0	1
*Ae. pembaensis*	0	1	0	0	0	0	1
*An. gambiae* (Giles, 1902)	0	0	2	0	0	0	2
*Cx. annulioris*	0	0	1	0	0	0	1
*Cx. quinquefasciatus*	253	1,136	715	211	3	46	2,364
*Cx. rubinotus* (Theobald)	0	0	0	0	6	0	6
*Cx. univittatus* (Theobald, 1901)	1	2	2	0	0	0	5
*Cx. zombaensis*	16	8	7	12	0	0	43
Total (mean ± SD)	277 (9.2 ± 10.0)	1,156 (28.9 ± 29.4)	737 (18.4 ± 38.1)	529 (19.6 ±36.7)	455 (16.9 ± 19.9)	110 (4.1 ± 5.1)	3,264

**Table 5:** Blood meal sources of the blood-fed mosquitoes collected in Malindi, Kilifi and Mombasa urban sites in coastal Kenya from November 2016 to April 2017

Species	Site	Location	No. tested	Human (%)	Bovine (%)	Goat (%)	Chicken (%)	Unidentified (%)
*Ae. aegypti*	Kilifi	Outdoor	4	3 (75)	0 (0)	0 (0)	0 (0)	1 (25)
		Indoor	1	0 (0)	0 (0)	0 (0)	0 (0)	1 (100)
	Malindi	Outdoor	9	0 (0)	0 (0)	0 (0)	0 (0)	9 (100)
	Mombasa	Indoor	1	1 (100)	0 (0)	0 (0)	0 (0)	0 (0)
*Cx. quinquefasciatus*	Kilifi	Outdoor	7	2 (28.6)	0 (0)	0 (0)	2 (28.6)	3 (42.9)
		Indoor	19	11(57.9)	0 (0)	0 (0)	1 (5.3)	7 (36.8)
	Malindi	Indoor	73	22(30.1)	0 (0)	0 (0)	3 (4.1)	48 (65.8)
	Mombasa	Outdoor	1	1 (100)	0 (0)	0 (0)	0 (0)	0 (0)
		Indoor	43	7 (16.3)	0 (0)	0 (0)	0 (0)	36 (83.7)
*Cx. univittatus*	Malindi	Indoor	1	0 (0)	0 (0)	0 (0)	0 (0)	1 (100)
	Mombasa	Indoor	2	1 (50)	0 (0)	0 (0)	0 (0)	1 (50)
Total			161	48(29.8)	0 (0)	0 (0)	6 (3.7)	107(66.5)

**Table 6:** Total number of mosquito pools tested and the proportion positive for *Flavivirus* in the Kilifi, Mombasa and Malindi urban sites in coastal Kenya from Nov. 2016 to April 2017

Species	Number of pools (positive)					
	Site	Sex	Biogents sentinel traps	Light traps	Larvae	Soil samples
*Ae. aegypti*	Kilifi	F	8 (3)	1 (1)	5 (1)	5 (0)
		M	14 (5)	4 (3)	6 (2)	8 (3)
	Malindi	F	13 (3)	3 (0)	6 (0)	6 (2)
		M	11 (0)	2 (0)	7 (0)	4 (0)
	Mombasa	F	4 (1)	3 (0)	5 (0)	0 (0)
		M	3 (0)	3 (1)	8 (1)	0 (0)
	Sub total		53 (12)	16 (5)	37 (4)	23 (5)
*Cx. quinquefasciatus*	Kilifi	F	6 (1)	9 (0)	4 (0)	0 (0)
		M	8 (0)	6 (0)	2 (0)	0 (0)
	Malindi	F	1 (0)	35 (3)	0 (0)	0 (0)
		M	2 (0)	17 (0)	0 (0)	0 (0)
	Mombasa	F	2 (0)	23 (0)	1 (0)	0 (0)
		M	3 (0)	10 (0)	1 (0)	0 (0)
	Sub total		22 (1)	100 (3)	8 (0)	0 (0)
	Grand total		75 (13)	116 (8)	45 (4)	23 (5)

## Data Availability

The supporting data is under the custodianship of the KEMRI-Wellcome Trust Data Governance Committee and is accessible upon request addressed to that committee.
